# Empiric outpatient antibiotic prescribing patterns in the emergency department

**DOI:** 10.1017/ash.2026.10376

**Published:** 2026-05-11

**Authors:** Apoorvjyot Badwal, Brittany Buffone, Cindy San, Victor Leung, Colin Lee

**Affiliations:** 1 Pharmacy, Fraser Health Authority, Surrey, Canada; 2 Providence Health Care, Canada

## Abstract

**Purpose:**

Inappropriate antimicrobial use in outpatient settings, such as Emergency Departments (EDs), is associated with increased risk of morbidity, costs, and antibiotic resistance. Prescribing patterns and guideline concordance in the ED is not well described in the literature. The primary objective of this study was to describe the empiric outpatient antibiotic prescribing patterns for frequent community-acquired infections seen in the ED at a large tertiary care teaching hospital. The secondary objectives were to evaluate concordance of antimicrobial prescribing with local guidelines, quantify the proportion of patients requiring antibiotic modification based on culture results, and 30-day ED revisits for the same infection.

**Methods:**

A retrospective chart review was conducted on a convenience sample of 200 adult patients discharged from the ED between October 1, 2022 to July 18, 2023. Data was analyzed to determine prescribing patterns and guideline concordance.

**Results:**

The most common infectious syndrome observed was skin and soft tissue infections (SSTIs). Overall, discordance was observed in 80% of cases, with 100% discordance seen with the treatment of COPD exacerbation and mild purulent SSTIs. Guideline discordance was predominantly due to treatment duration (67%) and antimicrobial selection (43%). Culture-directed therapy modifications were minimal (5%). There was no significant difference between antimicrobial concordance and therapy modification or between overall guideline concordance and 30-day revisit rates.

**Conclusion:**

Outpatient antimicrobial prescribing in the ED remains suboptimal despite available treatment guidelines, particularly in skin and respiratory infections. Discordance, mainly driven by extended treatment durations, highlights the need for improved Antimicrobial Stewardship practices in ED settings.

## Introduction

Traditionally, emergency departments (EDs) were designed to treat emergencies and transfer antibiotic care to the admitting teams following diagnostic investigations and cultures. The development of healthcare over the past decades has transitioned EDs into a primary point of care and acute ambulatory care setting that adopts antimicrobial prescribing responsibilities. With this transition, the role of Antimicrobial Stewardship in these settings remains largely unclear.^1,2^ According to the Center for Disease Control and Prevention (CDC), approximately half of all outpatient antimicrobial prescribing in the United States in 2016 was inappropriate based on selection, dose, or duration^3^ while in 2024, approximately 28% of all antibiotics prescribed were unnecessary.^4^ Furthermore, the improper use of antimicrobial agents has been associated with increased morbidity, costs, and antibiotic resistance rates.^2^

Effective Antimicrobial Stewardship programs implemented in inpatient settings are associated with better patient outcomes, reduced adverse effects, and decreased resistance rates.^5^ However, implementation of Antimicrobial Stewardship can be difficult in the ED due to the fast-paced environment, high turnover rates of clinicians, and high volume of patients.^6^ While the role of Antimicrobial Stewardship is well established in the inpatient setting, its role in the ED has not been established. Outpatient settings, including EDs, have been identified by the CDC as an area which needs improved Antimicrobial Stewardship interventions.^3^ With EDs being an area with high patient volume and various complicated and uncomplicated disease conditions, appropriate antimicrobial prescribing in the ED is essential to improve patient outcomes, decrease antibiotic resistance, and decrease risk of antimicrobial adverse events. Inappropriate antibiotic prescribing is one of the most modifiable risk factors that can contribute to the progression of resistance rates and is associated with treatment failure and poor outcomes.^5–7^

It is evident that antimicrobial prescribing in the ED may not be in concordance to national or site-specific guidelines. Therefore, the purpose of this study is to assess the empiric antibiotic prescribing patterns for the treatment of frequently seen community acquired infections in the ED of a tertiary care teaching hospital. The results of this study will contribute to current literature regarding appropriate antibiotic use and identify areas of guideline non-adherence in the ED when treating common outpatient infectious conditions.

## Methods

### Study design

This retrospective chart review included adults 19 years or older who visited the ED of an approximately 400-bed tertiary care teaching hospital that services inner city and vulnerable patient populations in Vancouver, British Columbia, Canada between October 1, 2022 and July 18, 2023, and who were discharged from the ED with a prescription for oral antibiotics. A point prevalence of 50 participants was included from the months of January, April, June/July, and October to account for seasonality of antimicrobial prescribing patterns. Patients were excluded if they were pregnant, immunocompromised, required inpatient admission, using concurrent antibiotics for a different indication, had recurrent infections, used antimicrobials in the last 30 days, self-discharged from the ED, received IV antibiotics for greater than 24 hours, referred for outpatient parenteral antimicrobial therapy, or being followed by Infectious Diseases consulting service. These inclusion and exclusion criteria helped select for a population that would qualify for first line standard empiric antibiotic regimens for uncomplicated community acquired infections.

### Data collection

A patient list was generated from the hospital’s electronic medical records to identify all patients who were prescribed oral antibiotics in the ED during the allotted study period. Within each period of January, April, June/July, and October, the patient list was randomized and the first 50 patients who met the inclusion/exclusion criteria were included. This study was approved by ethics review. Baseline characteristics collected included sex, age, antimicrobial allergy/intolerance status, and comorbidities. Additionally, culture sensitivities were collected if available.

### Outcome measures

The primary outcome was to characterize the empiric outpatient antimicrobial prescribing patterns for the treatment of common community acquired infectious diseases seen in the ED based on the frequency of empiric antibiotic agents selected and the duration of therapy. The secondary outcomes were to assess prescriber’s adherence of antimicrobial agent, dose, frequency, and duration to local prescribing guidelines using Firstline App.^8^ Concordance was assessed if local guidelines for that infectious condition were available. Overall discordance was defined as having an antibiotic agent, dose, frequency, or duration that differed from local guideline recommendations. Additionally, the proportion of patients contacted for culture directed change in therapy and the rates of readmission to the ED within 30 days for the same infection were assessed in guideline adherent versus non-adherent groups.

### Data analysis

Descriptive analysis was performed on demographic data and the primary outcome. For secondary outcomes, differences in proportions between guideline adherent and non-adherent groups for culture directed change in therapy and ED readmissions were compared using Fisher’s exact test where a *P* value of ≤.05 was statistically significant.

## Results

A total of 350 patients were screened, with 200 patients included in the study. Patient baseline characteristics are summarized in Table 1. The average age of the cohort was 45 years, and 50% were female. The most common infectious syndrome observed was skin and soft tissue infections (SSTIs) at 39%, followed by genitourinary infections at 25%, and respiratory infections at 21%. The most frequently reported antimicrobial allergy was penicillin at 9.5%. A total of 63 cultures were collected, the most common being urine cultures (49%).

**Table 1.** Baseline characteristics

Baseline characteristic	Overall (n = 200)
Mean age (years)	45 (Range: 20–94)
Sex	
Female (%)	100 (50)
Male (%)	100 (50)
Antimicrobial allergy/intolerance	
Penicillins (%)	19 (9.5)
Sulfa (%)	12 (6)
Fluoroquinolones (%)	5 (2.5)
Macrolides (%)	5 (2.5)
Other (%)	7 (3.5)
Source of infection	
Skin and soft tissue (%)	78 (39)
Genitourinary (%)	50 (25)
Respiratory (%)	41 (21)
Head, eyes, ears, nose, throat (%)	13 (6)
Gastrointestinal/intra-abdominal (%)	12 (6)
Sexually transmitted (%)	2 (1)
Other (%)	4 (2)
Number of cultures collected (n = 63)	
Urine (%)	31 (49)
Blood (%)	11 (17)
Genital (%)	8 (13)
Wound (%)	6 (10)
Other (%)	7 (11)

The most commonly prescribed antimicrobial classes were penicillins and cephalosporins (Table 2). The median duration of antimicrobials for most disease states was 7 days or longer, with the shortest duration of therapy seen with community-acquired pneumonia (CAP) for a median of 5 days, while the longest duration of therapy was seen with the treatment of pelvic inflammatory disease for a median of 14 days.

**Table 2.** Empiric antibiotics and duration for common infectious indications

Antimicrobial prescribed (n = total number of antibiotics prescribed per disease state)	N (%)	Median duration (days, range)	Mean duration (days)
Community acquired			
Pneumonia (n = 21)		*5 (5–10)*	*6.1*
Penicillins	10 (47.6)	5 (5–7)	5.8
Macrolides	6 (28.6)	5 (5–10)	5.8
Cephalosporins	2 (9.5)	6 (5–7)	6
Tetracyclines	2 (9.5)	7	7
Fluoroquinolones	1 (4.8)	10	10
COPD exacerbation (n = 9)		*7 (4–14)*	*7.2*
Penicillins	4 (44.4)	7 (5–7)	6.5
Tetracyclines	3 (33.3)	7 (7–14)	9.3
Macrolides	1 (11.1)	4	4
Fluoroquinolones	1 (11.1)	7	7
Diverticulitis (n = 6)		*8.5 (7–10)*	*8.5*
Penicillins	3 (50.0)	7 (7–10)	8
Fluoroquinolones	2 (33.3)	8.5 (7–10)	8.5
Metronidazole	1 (16.7)	10	10
Mild non-purulent cellulitis (n = 68)		*7 (3–14)*	*6.7*
Cephalosporins	45 (66.2)	7 (3–8)	*6.4*
Cotrimoxazole	10 (14.7)	7	7
Tetracyclines	6 (8.8)	7 (7–14)	8.2
Penicillins	4 (5.9)	7 (5–7)	6.5
Clindamycin	3 (4.4)	7 (5–7)	6.3
Mild purulent cellulitis (n = 23)		*7 (5–10)*	*7.1*
Cephalosporins	9 (39.1%)	7 (7–10)	7.3
Cotrimoxazole	5 (21.7%)	7 (7–10)	7.6
Tetracyclines	4 (17.4%)	7	7
Penicillins	3 (13.0%)	7	7
Metronidazole	1 (4.3%)	5	5
Fluoroquinolones	1 (4.3%)	5	5
Cystitis (n = 25)		*7 (1–7)*	*5.6*
Nitrofurantoin	17 (68.0%)	7 (5–7)	6.4
Cotrimoxazole	3 (12.0%)	3 (3–7)	4.3
Fosfomycin	2 (8.0%)	1	1
Fluoroquinolones	2 (8.0%)	5 (3–7)	5
Cephalosporins	1 (4.0%)	7	7
Pyelonephritis (n = 15)		*7 (7–10)*	*7.2*
Cephalosporins	14 (93.3%)	7 (7–10)	7.2
Fluoroquinolones	1 (6.7%)	7	7

Of the 200 patients, 42 were given a diagnosis of infections that did not have corresponding local treatment guidelines and therefore could not be assessed for guideline concordance. Of the patients evaluated for guidelines concordance, 126/158 (80%) were discordant by either agent, dose, frequency, or duration, with 100% discordance seen when treating COPD exacerbations or mild purulent cellulitis (Table 3). Conversely, pelvic inflammatory disease had the lowest rate of discordance at 0%.

**Table 3.** Guideline discordance among disease states

	Number of Discordant Cases	Discordance (%)
**Overall discordance**	126/158	80
**Discordance by disease state** ^1^		
COPD exacerbation	9/9	100
Mild purulent cellulitis	18/18	100
Community acquired pneumonia	19/20	95
Mild non-purulent cellulitis	57/60	95
Diverticulitis	3/4	75
Cystitis	16/25	64
Chlamydia	1/2	50
H. Pylori	1/2	50
Pyelonephritis	2/15	13
Pelvic inflammatory disease	0/3	0

^1^Discordance for each disease state was determined using local site-specific guidelines on Firstline App – Providence Health Care.^8^

**Figure 1.**
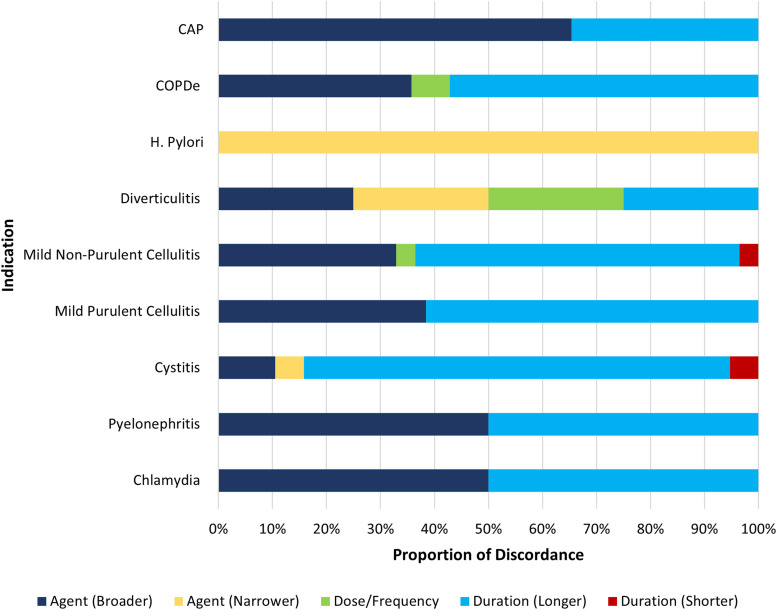
Reason for discordance by indication.

Discordance was primarily due to prolonged duration (67%), followed by overly broad antimicrobial agent (41%), and dose or frequency (6%) (Figure 1). In 87% of discordant cases (110/126), the rationale for discordance was not documented by the prescriber. Among documented rationales, concern for the risk of methicillin-resistant Staphylococcus aureus (MRSA) was most common (8%), followed by antibiotic allergy (1.6%), need for additional coverage (1.6%), patient adherence concerns (0.8%), and breastfeeding (0.8%).

Out of the 158 patients being evaluated for guideline concordance, 61 were followed up with via telephone within 72 hours of initial antibiotic prescription from the ED. Empiric antibiotic therapy was modified due to culture results in 3 of those patients, with changes primarily due to inadequate empiric coverage in 2 patients and MRSA culture in 1 patient. There was no significant association between initial antimicrobial prescribing concordance and subsequent change in therapy (*P = .78*). Overall, 25% (40/158) of patients revisited the ED within 30 days for the same condition (Table 4). Revisit rates were not significantly different between guideline concordance and discordance cases (*P = .50*).

**Table 4.** ED revisits in 30 days

	Discordant cases	Concordant cases
**Did not revisit ED in 30 days**	92	26
**Revisited ED in 30 days**	34	6
**Reason for revisit**	(%)	(%)
Worsening infection	17 (50)	3 (50)
Antibiotic allergy	0 (0)	1 (16.7)
Misplaced original prescription	5 (14.7)	0 (0)
Further investigations	5 (14.7)	2 (33.3)
Noncompliant	4 (11.8)	0 (0)
Other	3 (8.8)	0 (0)

## Discussion

The findings of this study highlight significant discordance in antimicrobial prescribing patterns despite the availability of guidelines in the ED of a large tertiary care teaching hospital, with an overall discordance of 80%. This is consistent with previous studies indicating high rates of discordance in ED empiric antimicrobial prescribing.^2,9–12^ While the predominant use of penicillins and cephalosporins aligns with common treatment guidelines, the high rate of discordance suggests suboptimal prescribing practices. The primary driver of discordance was prolonged therapy duration beyond recommended guidelines. For example, local guidelines recommend a 5-day course for cephalosporins for cellulitis and nitrofurantoin for UTI, yet a median duration of 7 days was prescribed. This may be attributed to Computerized Physician Order Entry (CPOE), which provides a default duration of 7 days for most antimicrobials. This can be optimized by systematizing antimicrobial durations by specific conditions or by shortening the default duration to 5 days and automating alerts for reassessment of therapy.

The study overlapping with the COVID-19 pandemic may have influenced case capture and outcomes due to major shifts in healthcare utilization. During this period, decreased ED visits may have resulted in a greater proportion of higher acuity patients. The CDC reports from 2019 to 2020, there was a 25% decrease in outpatient prescriptions due to fewer ED visits.^13^ In addition, healthcare systems experienced changes in workflow and staffing pressures that may have influenced clinical approach and prescribing practices due to uncertainty around changing protocols.

This study identified COPD exacerbations and SSTIs as having the highest discordance rates. These findings mirror previous research showing significant discordance in the treatment of SSTIs in ED, where up to 71% of patients received antibiotics discordant with the Infectious Disease Society of America (IDSA) guidelines.^11^ Another retrospective study reported that only 39% of patients who were admitted to the ED with SSTI received guideline directed therapy.^12^ A possible reason for high discordance rates in SSTIs is its difficulty to diagnose, leading to variation in treatment approach.^10^

The ED is a unique practice area which has high acuity cases with high patient turnover, further complicating the implementation of effective antimicrobial stewardship practices. A notable finding in this study was the lack of documented rationale for discordance in 87% of the cases. This may be attributed to the setting of the ED being fast paced with high patient volume. However, this gap in documentation can hinder efforts to address underlying reasons behind non-adherence to guidelines. Moreover, inappropriate antibiotic prescribing is one of the most modifiable risk factors that can contribute to the progression of resistance rates,^5^ which is why it is essential for appropriate documentation by prescribers. Previous studies have shown that the role of pharmacists in the ED have significantly improved antimicrobial stewardship practices. A retrospective cohort study which looked at patients who were admitted to the ED found that for patients diagnosed with intraabdominal infections, guideline-based empiric therapy was implemented 62% of the time in the presence of a pharmacist compared to 44% of the time in the absence of a pharmacist.^14^ These findings suggest that similar interventions could be beneficial at our hospital to improve guideline concordance. In addition, one of the identified barriers to effective antimicrobial stewardship in the ED is the lack of diagnostic clarity.^1^ Approximately 41% of patients have pending laboratory and microbiological data at the time of discharge from ED, which may lead to diagnostic uncertainty.^5^ This uncertainty may lead clinicians to prescribe more broadly, which may lead to overutilization of antibiotics.^15^ This is also observed in this study where 96% of discordant agent cases involved unnecessarily broad coverage for *Pseudomonas*, MRSA, and atypical pathogens. This finding raises concerns about the potential for increasing antimicrobial resistance and questions whether overly broad empiric antimicrobials are appropriate for treating uncomplicated cases in the ED.

This study found no statistical significance between rates of ED revisit within 30 days of the same infection and antimicrobial concordance. Although revisitation rate was higher in the discordant group, 50% of revisits in both groups were due to worsening infection. These findings suggest that adherence to guideline therapy alone may not determine outcomes and factors such as patient presentation and clinical judgment will influence prescribing decisions.

### Limitations

This study has several limitations. First, the use of convenience sampling introduces selection bias that may not represent the general population. In addition, it was retrospective, conducted at a single inner-city center, and excluded pregnant and immunocompromised populations, which limits generalizability. Moreover, not all disease states had corresponding local guidelines to assess concordance, leading to their exclusion from the secondary analysis. The diagnoses documented could also not be confirmed due the retrospective chart review. Lastly, the study did not determine adherence to prescribed treatment regimens, which may confound the evaluation of patient outcomes.

### Clinical and research implications

This study underscores significant challenges in antimicrobial prescribing practices within the ED of a large tertiary care teaching hospital. Despite the availability of treatment guidelines, the high incidence of discordance observed highlights a critical area for improvement in optimizing patient care and combating antimicrobial resistance. Addressing these challenges requires multifaceted strategies. Enhanced education and training for healthcare providers, coupled with refinements in CPOE systems, could facilitate adherence to evidence-based guidelines. Moreover, the involvement of clinical pharmacists in antimicrobial stewardship initiatives could play a pivotal role in promoting guideline-concordant prescribing and improving patient outcomes. The results from this study highlights the need for further development and refinement of local treatment guidelines, particularly for conditions lacking specific recommendations. Additionally, further research is needed in other practice areas such as inpatient settings and primary care clinics to compare rates of discordance.

This study offers insight into empiric outpatient antimicrobial prescribing patterns of common community-acquired infections seen in the ED of a tertiary care site, ultimately revealing a high incidence of antimicrobial discordance, driven primarily by prolonged duration and broad antibiotic use. By addressing the challenges in prescribing practices and implementing effective stewardship practices, healthcare institutions can mitigate the risks associated with inappropriate antimicrobial use, preserve the efficacy of antibiotics, and enhance patient safety and outcomes in outpatient populations.
